# Recurrent Subdural and Epidural Hematomas: A Case Report of Complications Following Craniotomy and Middle Meningeal Artery Embolization

**DOI:** 10.7759/cureus.73445

**Published:** 2024-11-11

**Authors:** Jennifer Ngandu, Riddhi Chaudhari, Barbara Fontenelle, Samer Kholoki

**Affiliations:** 1 Medical School, Saint James School of Medicine, Park Ridge, USA; 2 Internal Medicine, UChicago Medicine AdventHealth, La Grange, USA

**Keywords:** chronic subdural hematoma, craniotomy, epidural hematoma, middle meningeal artery embolization, subdural hematoma

## Abstract

This report presents a 76-year-old male patient who developed indolent right-sided upper and lower extremity weakness and low back pain following a fall that resulted in no trauma and was not prompted by syncope or vertigo. Imaging revealed a chronic subdural hematoma (cSDH) with midline shift, for which the patient underwent craniotomy and middle meningeal artery (MMA) embolization. Despite initial intervention, the patient experienced rare and severe complications, including the recurrence of a subdural hematoma (SDH), the development of an epidural hematoma, and frontal lobe herniation.

The significance of this report lies in its complications following MMA embolization, a treatment, though novel, generally considered safe and effective for cSDH. This case highlights the potential for unexpected and severe, life-threatening outcomes in certain patients, emphasizing the need for physicians to remain vigilant for such complications. By exploring these rare occurrences, this report contributes to the evolving understanding of embolization risks and the need for structuring treatment strategies tailored to patients based on vulnerability.

## Introduction

Subdural hematomas (SDHs) present in three distinct forms, acute, subacute, and chronic, each with unique clinical characteristics and implications [1]. Acute SDHs typically arise from significant head trauma, with symptoms manifesting rapidly, often within minutes to hours. In contrast, subacute SDHs may develop over hours to days, frequently following a concussion. Chronic SDHs (cSDHs), however, are more insidious, commonly affecting individuals over the age of 65, with symptoms emerging gradually over weeks to months following seemingly minor head trauma [1].

The clinical presentation of cSDHs can be subtle, in stark contrast to the dramatic "worst headache of my life" often reported in subarachnoid hemorrhage (SAH) [2]. This subtlety, as highlighted in the present case, underscores the potentially life-threatening nature of cSDHs, particularly when diagnosis and treatment are delayed. SDHs contribute to approximately 25% of fatalities resulting from traumatic head injuries, with the elderly population being especially vulnerable due to age-related brain atrophy, which increases the tension on bridging veins and predisposes them to rupture [1]. Statistically, there exists an annual incidence of 14-20 per 100,000 individuals. It is expected to become the most common neurosurgical emergency in the near future [3]. The disease has an impact on life expectancy, with a one-year standardized mortality ratio increased across all age groups. Morbidity and mortality rates are 11% and 4%, respectively [3]. With surgical intervention being the standard treatment, there remains a high recurrence risk, affecting about 10% of surgically treated patients [3].

The management of SDHs typically necessitates surgical intervention, with the choice of procedure, either craniotomy or burr hole surgery, depending on the severity and characteristics of the hematoma [4]. Craniotomy involves the surgical removal of the hematoma through suction, whereas burr hole surgery entails draining the hematoma through a small hole drilled in the skull [4]. While most cases of cSDH can be effectively treated with surgery, the risk of recurrence is a significant concern. [5]. Recurrent hematomas can occur in up to 20% of patients after surgical evacuation, often requiring additional interventions [6].

This case report presents a complex scenario involving recurrent subdural and epidural hematomas following craniotomy and embolization in a 76-year-old patient. The recurrence of both subdural and epidural hematoma in the same patient is a rare and challenging occurrence, often leading to unexpected complications that necessitate further surgical intervention and prolonged hospitalization. This report aims to highlight the diagnostic challenges, management strategies, and potential complications associated with recurrent hematomas, emphasizing the importance of vigilant monitoring and a multidisciplinary approach in such complex cases.

## Case presentation

A 76-year-old male patient with a medical history significant for hypertension, hyperlipidemia, and chronic obstructive pulmonary disease presented to the emergency department with a three-week history of progressive right-sided upper and lower extremity weakness, unsteady gait, and chronic lower back pain. The symptoms began following a slip, with no immediate trauma reported. The patient endorsed a gradual aggravation of right-sided weakness, accompanied by tremors and muscle spasms, starting at the right hip and radiating down the right leg. He denied any associated pain, paresthesia, visual disturbances, speech difficulties, or anticoagulant use. The patient, who has a history of chronic low back pain due to degenerative disc disease and maintains a physical activity routine of stationary cycling five times a week, reported increasing difficulty in performing his exercises due to his symptoms.

Physical exam on admission demonstrated right-sided upper and lower extremity weakness, with motor strength rated 3/5 on the right and 5/5 on the left. The patient was alert, oriented, and hemodynamically stable, with vital signs within normal limits.

**Figure 1:**
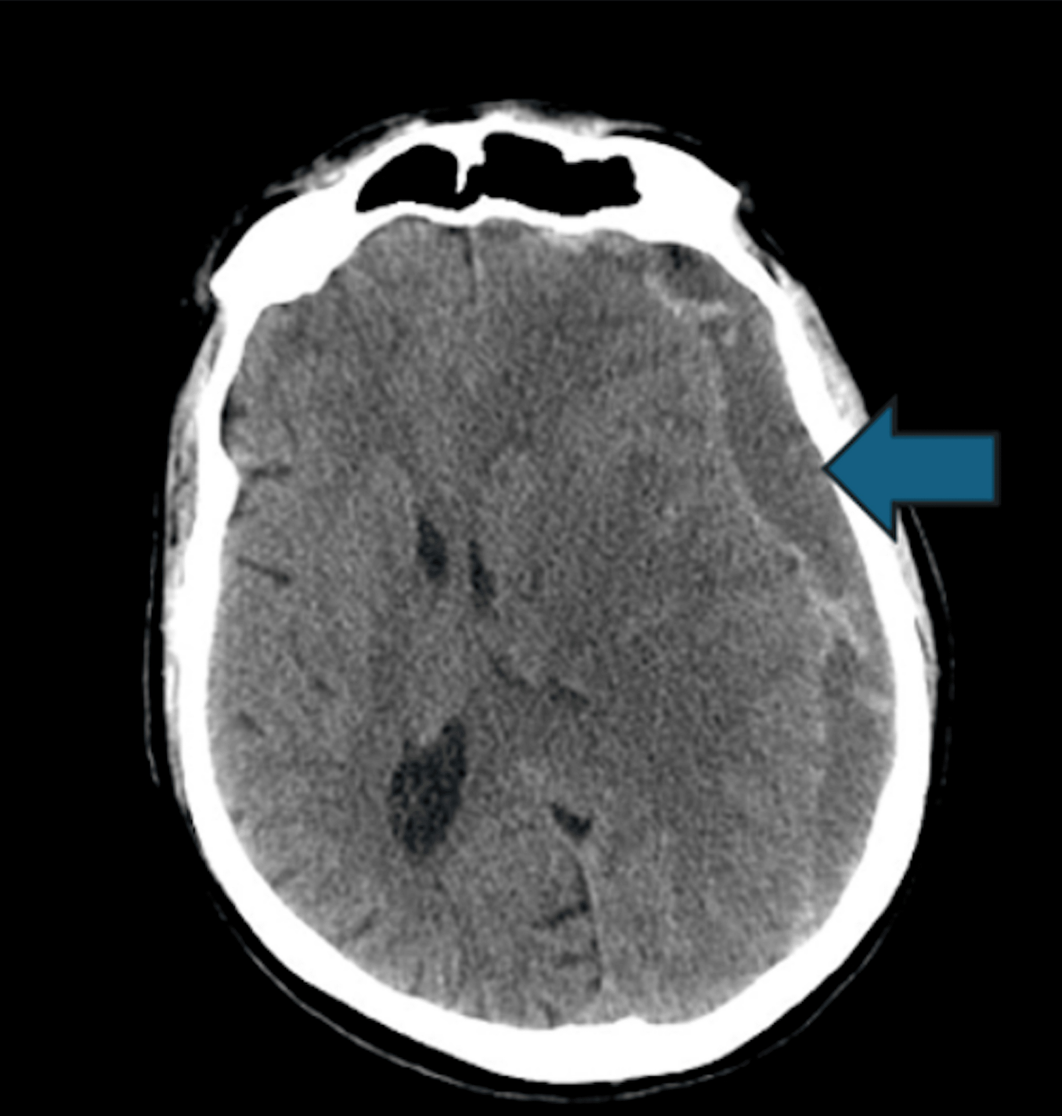
Acute and subacute left frontoparietal subdural hematoma with mass effect and midline shift to the right The arrow indicates the location of the subdural hematoma.

Initial lumbosacral CT showed degenerative changes consistent with the patient's history, including L3-L4 spinal stenosis. A head CT without contrast revealed a large subacute and chronic SDH in the left frontoparietal region, measuring 3 cm in thickness, with mass effect on the lateral ventricles, a 1.6 cm midline shift, and evidence of suspected subfalcine herniation to the right (Figure 1).

**Figure 2:**
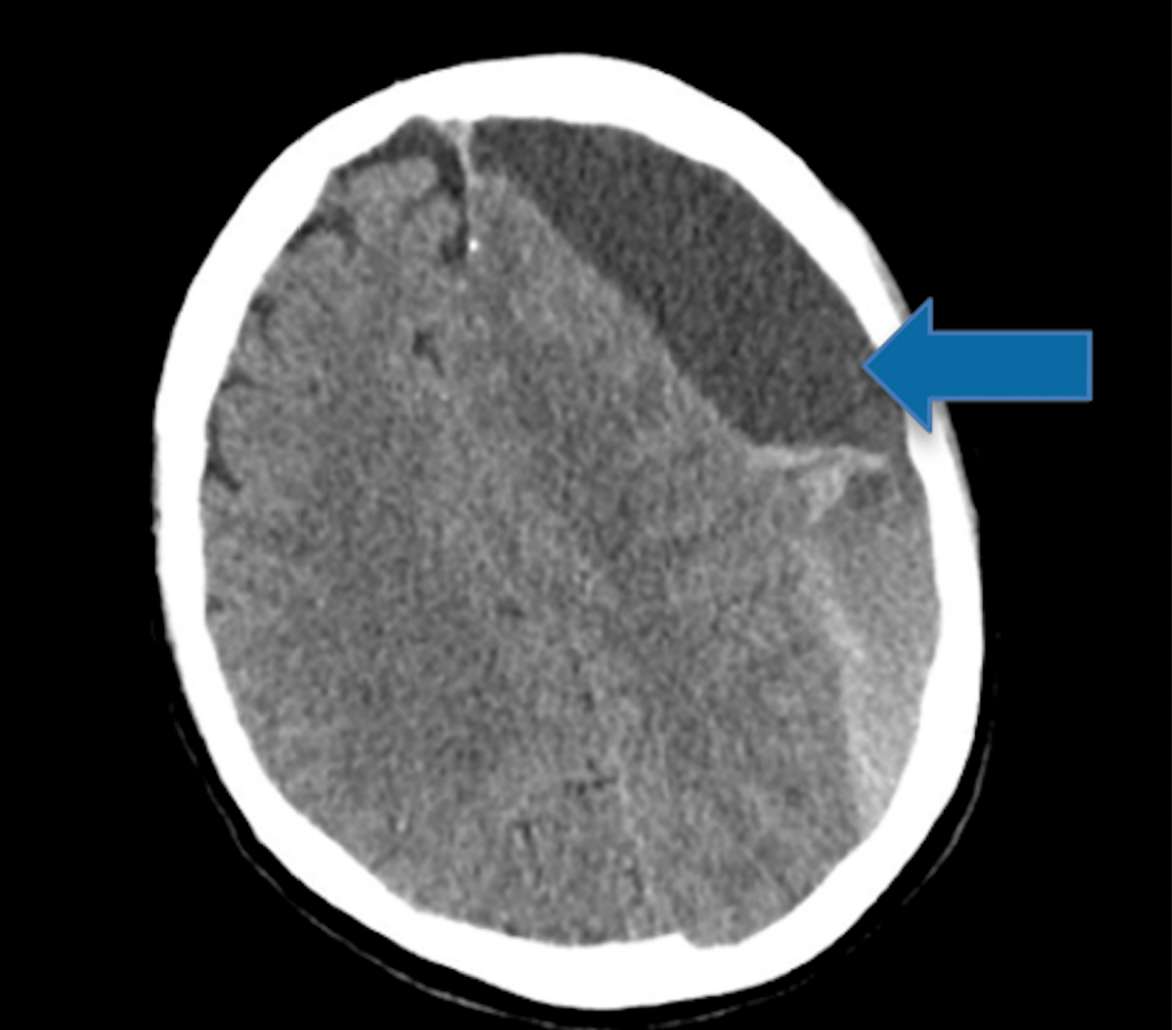
Increase in the size of the subacute subdural hematoma of the left frontoparietal region with mass effect and suspected subfalcine herniation The arrow indicates an increase in the size of the subdural hematoma.

The patient was scheduled to undergo a left frontotemporal craniotomy with membrane lysis to evacuate the hematoma on the second day of admission. A preoperative head CT on the day of surgery revealed an increase in hematoma size to 3.3 cm in thickness (previously 3 cm), with the midline shift remaining at 1.6 cm (Figure 2).

**Figure 3:**
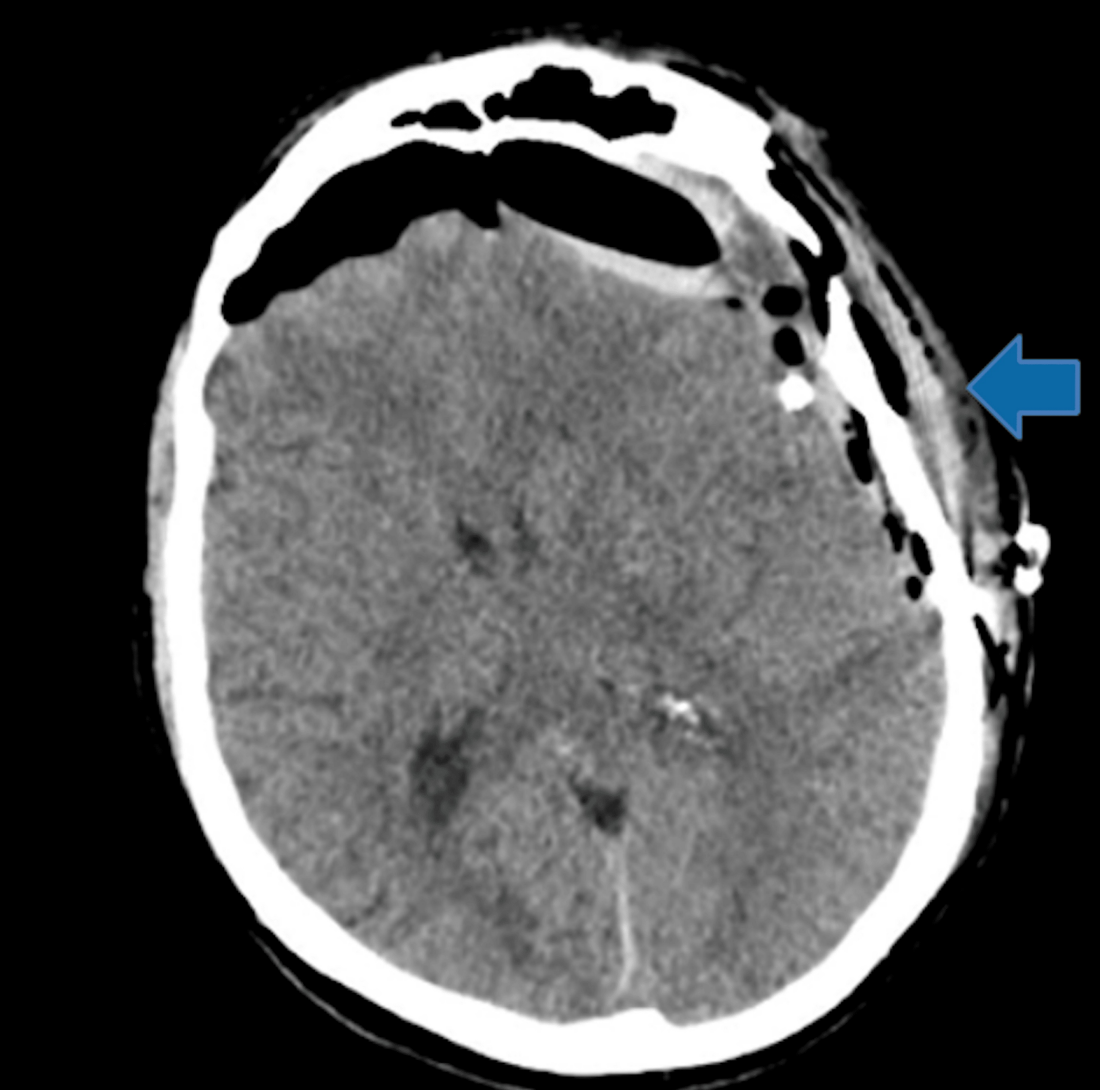
Postoperative imaging showing evacuated left subdural hematoma following craniotomy The arrow indicates the evacuated subdural hematoma.

The patient underwent several burr holes in the frontal, parietal, and temporal regions with a left frontotemporal craniotomy and membrane lysis, followed by planned prophylactic middle meningeal artery (MMA) embolization to minimize the risk of rebleed. During the craniotomy, oil-like fluid was noted upon the dural opening, followed by the evacuation of a subdural clot, coagulation of bridging veins, cauterization of membranes, and placement of a ventricular catheter in the subdural space. A postoperative head CT without contrast, performed the following day, confirmed the successful evacuation of the left subdural region and a reduction in midline shift from 1.6 cm to 0.9 cm (Figure 3). 

Three days post-craniotomy, the patient underwent a cerebral angiogram followed by the planned embolization of the left MMA. Before the embolization, the patient reported persistent headaches; physical examination demonstrated right-sided weakness, no cranial nerve deficits, and 5/5 strength in all extremities. The angiogram was performed via a right common femoral artery approach, puncturing and cannulating using a 5F micropuncture. A 6F arterial sheath was placed over a guidewire and attached to heparinized saline into the aortic arch. The following vessels were catheterized: left common carotid artery, left internal carotid artery, left external carotid artery, right common carotid artery, right internal carotid artery, right external carotid artery, and right common iliac artery. All vessels were patent with the absence of aneurysm, vascular malformation, or hemorrhage. A diagnostic catheter (Echelon 14 microcatheter) was then positioned in the left MMA, advanced proximal to its bifurcation, and embolization was performed using polyvinyl alcohol (PVA) particles (150-250 microns). A subsequent angiogram confirmed stagnant flow in the frontoparietal and squamosal MMA branches, along with a reduction in the size of the more distant branch vessels. 

Twenty-four hours post-procedure, headaches were rated at 3/10 in intensity with the presence of hypokalemia, hypocalcemia, anemia, and mild hyperglycemia. The patient denied focal weakness, numbness, tingling, or dizziness. Three days post-procedure, the patient underwent a head CT without contrast which demonstrated no evidence of active extravasation, aneurysm, or hemorrhage. The patient was discharged home with headaches managed with analgesics and no reports of numbness, tingling, nausea, or dizziness. There remained a trace of right-sided weakness. His condition at discharge was noted as fair, and he was transferred from the hospital to a rehabilitation facility.

On the fourth day of rehab, the patient was transferred to the ICU due to new-onset word-finding difficulty and right-sided weakness, prompting readmission to the hospital. Upon evaluation, he exhibited slurred speech, right upper extremity weakness, and a left-sided headache. Motor strength was 5/5 in the left upper and lower extremities and 3/5 in the right upper and lower extremities. A head CT without contrast demonstrated an SDH overlying the left cerebral convexity, measuring 2.6 cm with midline shift and an epidural hematoma. The patient underwent an additional craniotomy for recurrent left SDH and epidural hematoma.

**Figure 4:**
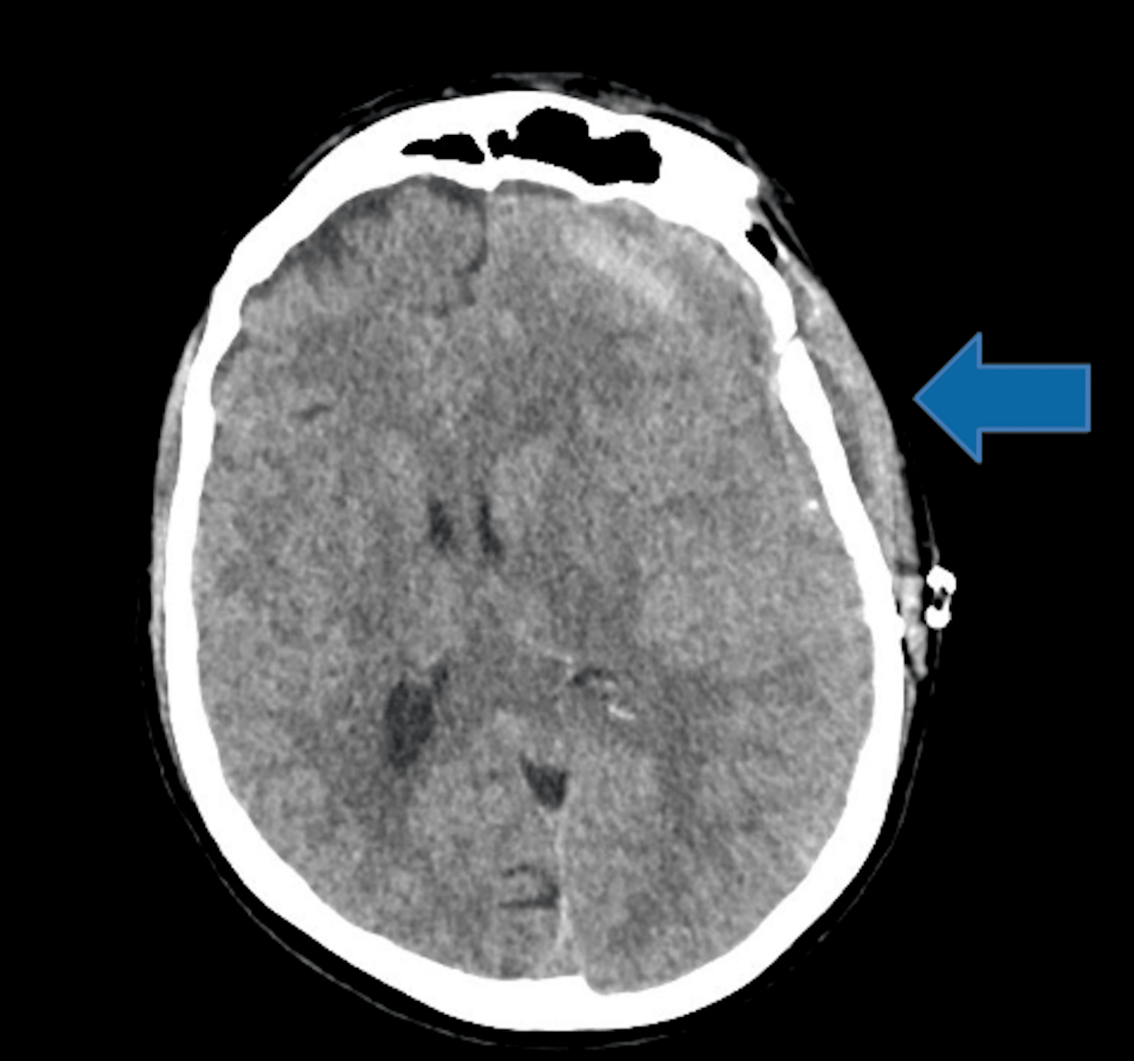
Postoperative imaging after middle meningeal artery embolization for recurrent subdural hematoma and epidural hematoma The arrow indicates the location of evacuated recurrent subdural and epidural hematomas.

On postoperative day 2, the patient underwent a head CT without contrast which demonstrated a persistent midline shift measuring 0.7 cm with a decrease in pneumocephalus (Figure 4).

**Figure 5:**
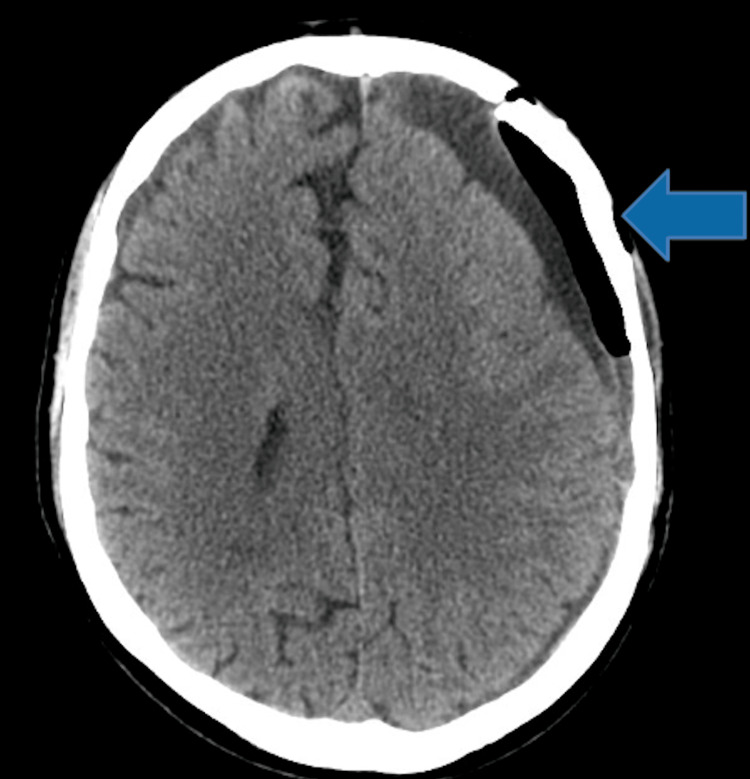
Day 11 postoperative findings: stable 0.7 cm left to right midline shift The arrow indicates the location of evacuated recurrent subdural and epidural hematomas, 11 days post-craniotomy.

Eleven days postoperatively, the patient was discharged with improved headaches, continued expressive aphasia, and hemodynamic stability (Figure 5).

## Discussion

SDHs are a critical and potentially fatal type of intracranial hemorrhage, characterized by the accumulation of blood between the dura mater and the arachnoid membrane [7]. Although SDHs can present acutely following a significant head injury, cSDHs, which develop more gradually, pose a particularly insidious threat due to their slow and often deceptive progression [7].

The present case involving a 76-year-old male patient underscores the profound clinical risk associated with cSDHs. The subtle onset of symptoms, which can easily be mistaken for less severe conditions, highlights the importance of maintaining a high level of clinical suspicion. Timely recognition and intervention are essential to prevent potentially irreversible neurological damage or death when managing such cases.

This case is significant due to the rare and severe complications following MMA embolization, a procedure generally considered safe, though novel. The recurrence of the SDH, along with the development of an epidural hematoma and associated frontal lobe herniation, highlights the potential for life-threatening outcomes even after standard interventions as experienced by this patient.

cSDHs are more commonly seen in individuals over the age of 65, typically following injuries that may appear minor, with symptoms developing gradually [8]. These hematomas often result from damage to bridging veins, with an increased risk of rebleeding if another injury occurs [9]. While the origin is primarily venous, an alternative pathophysiology suggests that injury to dural border cells leads to inflammation, promoting blood vessel formation and fibrogenesis [10]. This process results in the development of a new dural layer composed of two layers, with the external layer exacerbating the inflammatory response [10]. This layer contains fragile capillaries that are prone to injury, contributing to rebleeding and the progression of cSDH [10].

Management of cSDH typically depends on the severity of symptoms and the size of the hematoma. Conservative treatment with observation is often appropriate if there are no symptoms, but surgical evacuation is frequently necessary, which carries a risk of recurrence [11]. MMA embolization has emerged as a treatment option because it targets the blood supply to the dura mater and capillaries; embolization effectively resolves bleeding by blocking blood flow [11].

In 2017, Dr. Knopman from Weill Cornell was the first physician to perform MMA embolization as an alternative to surgical drainage for treating SDH, making this procedure a novel approach [12]. According to Dr. Knopman, the recurrence rates of SDHs have decreased from 15-20% to less than 4% with this innovative technique, which is particularly beneficial for patients presenting with significant headaches, weakness, or enlarging SDH or those who are contraindicated for surgical intervention [12].

In a comparison study, Ban et al. evaluated 72 patients who underwent MMA embolization, 27 of whom were asymptomatic and 45 symptomatic, against 469 patients who received conventional surgical treatment [8]. All 27 asymptomatic patients who underwent MMA embolization achieved resolution, with rebleeding occurring in only one out of the 45 symptomatic patients [8]. Overall, treatment failure was significantly lower in the embolization group (1.4%) compared to the conventional treatment group (27.5%) [8]. Supporting these findings, another study comparing burr hole craniotomy with MMA embolization in patients with previous surgical evacuation found a higher success rate with MMA embolization, with only one case of treatment failure compared to a 33.3% recurrence rate in those treated with burr hole craniotomy [13]. Overall, it is evident in the current literature that MMA embolization is an effective treatment for cSDH [8,14,13].

Management of recurrence has been documented to be successful via embolization, burr hole irrigation, or craniotomy, such as the present patient [15]. The following tables delineate current studies demonstrating successful and unsuccessful MMA embolization in the setting of SDH (Table 1 and Table 2).

**Table 1:** Case reports on successful SDH management via MMA embolization SDH: subdural hematoma; cSDH: chronic subdural hematoma; PVA: polyvinyl alcohol; NBCA: N-butyl cyanoacrylate; MMA: middle meningeal artery; UI: international unit

Study	Country	Age/sex	Description of procedure	Outcome
Xuan et al. [16]	USA	61/M	Embolization one day prior to twist-drill craniotomy with PVA particles mixed with heparin 500 UI	Successful embolization; the patient underwent a subsequent twist-drill craniotomy to remove the hematoma. At three-month follow-up, no clinical symptoms were found
Tempaku et al. [17]	Japan	91/F, 73/M, 87/M, 79/M, 85/M	Recurrent SDH treated with single burr hole surgery and MMA embolization with PVA particles	Promising potential as an alternative treatment for recurrent cSDH, with various substances like PVA particles, NBCA, coils, and gelatin sponge effectively inhibiting recurrence in reported cases
Kang et al. [18]	Korea	13/M	Recurrent SDH treated with burr hole trephination and MMA embolization performed with four coils	There has been no remarkable clinical finding during the five years of follow-up

**Table 2:** Case reports on unsuccessful SDH treatment via MMA embolization SDH: subdural hematoma; cSDH: chronic subdural hematoma; PVA: polyvinyl alcohol; MMA: middle meningeal artery

Study	Country	Age/sex	Description of procedure	Outcome
Otsuji et al. [19]	Japan	59/M	Endovascular embolization of the MMA using tris-acryl gelatin microspheres	Unsuccessful MMA embolization with the development of right cSDH with 15 mm thickness and midline shift, two months postoperatively, for which burr hole irrigation was conducted as treatment
Cristaldi et al. [20]		61/F	Left MMA embolization for cSDH	Facial nerve palsy after MMA embolization for cSDH
Chihara et al. [15]	Japan	59/M	Burr hole irrigation with the recurrence of SDH for which MMA embolization with PVA particles (250-355 um) and fibered coils was performed followed by burr hole irrigation	Recurrence occurred three months post-procedures for which craniotomy was performed to evacuate the hematoma
Present report	USA	76/M	Left frontotemporal craniotomy with membrane lysis, followed by prophylactic MMA embolization with PVA particles (150-250 microns)	Recurrence occurred four days postoperatively with SDH, epidural hematoma, and frontal lobe herniation for which craniotomy was performed

Despite these promising outcomes, it is crucial to acknowledge that MMA embolization, like any intervention, is not without risks. We report a rare cause of SDH recurrence and epidural hematoma development despite prior treatment with craniotomy and prophylactic MMA embolization. While MMA embolization represents a significant advancement in SDH management, the risk of adverse events necessitates continued vigilance and a balanced approach to treatment based on these findings.

Limited studies and case reports demonstrate the occurrence of epidural hematoma or recurrence of SDH as a complication following MMA embolization for the treatment of cSDH. While the procedure has been considered minimally invasive and as an alternative or adjunct to surgical evacuation, with a strong safety profile, the paucity of studies on epidural hematomas or SDHs linked to MMA embolization suggests that this complication is exceedingly rare in current clinical practice. This highlights the efficacy and safety of MMA embolization as a treatment option, particularly in patients who are unable to undergo surgical intervention; however, like the present case, risks are present. Continued monitoring is necessary to ensure that any potential rare complications are identified and addressed in future studies.

## Conclusions

MMA embolization using PVA particles has emerged as a promising intervention for managing refractory cSDH. This case underscores the efficacy of MMA embolization in reducing recurrence rates and offers an alternative to traditional surgical methods. However, the unexpected complications experienced by this 76-year-old patient, namely, the recurrence of SDH, development of an epidural hematoma, and subsequent frontal lobe herniation, highlight how MMA embolization is not devoid of significant risks. These findings emphasize the critical need for meticulous postoperative monitoring and the implementation of consistent imaging protocols to promptly identify and address potential complications. While current literature predominantly supports the safety and effectiveness of MMA embolization, this case illustrates that rare but severe adverse outcomes can occur, necessitating a cautious and individualized approach to patient selection and management.

Furthermore, this case contributes to the growing body of evidence that calls for comprehensive studies to better understand the risk factors and mechanisms underlying such complications. Future research should focus on identifying patient-specific vulnerabilities and refining embolization techniques to enhance safety profiles. Additionally, establishing standardized postoperative surveillance strategies will be essential in mitigating risks and improving patient outcomes.

In conclusion, while MMA embolization represents a significant advancement in the treatment of cSDH, healthcare providers must remain vigilant for atypical and life-threatening complications. A multidisciplinary approach, combining surgical expertise with interventional radiology, and tailored patient care protocols will be paramount in optimizing treatment efficacy and ensuring patient safety.
